# South Carolina Dental Association

**Published:** 1888-08

**Authors:** R. Atmar Smith

**Affiliations:** Rec. Secty. —*Southern Dental Journal*.


ARTICLE VII.

### SOUTH CAROLINA DENTAL ASSOCIATION.

GREENVILLE, JULY 24TH, 1888.

The Eighteenth Annual Meeting of the South Carolina State Dental Association was held at this place. commencing this morning. The first meeting was called to order in Dr. J. W. Norwood's office, but was adjourned to meet at Knights of Honor Hall, where the other sessions were held. The following members were present at this meeting: President, L. S. Wolf; Second vice-president, Brooks Rutledge; Corresponding Secretary, E. C. Ridgell; Recording Secretary, R. Atmar Smith; Treasurer, G. W. Dick; and Drs. B. H. Teague, D. L. Boozer, J. R. Thompson, J. T. Calvert, J. C. Oeland, A. P. Johnston, Theo. Johnston, J. M. Quattlebaum, B. J. Quattlebaum, J. W. Norwood, J. Q. McDavid, J. H. Alexander, W. A. Courtenay, G. F. S. Wright, P. B. Connor, D. R. McCallum.

The minutes of the Seventeenth Annual meeting were read and confirmed.

### RETIRING ADDRESS OF L. S. WOLF, PRESIDENT.

*Gentlemen* :—Now that I am about to retire from the position tendered me at our last meeting, permit me to acknowledge my sensibility of your kindness, and to thank you for the honor conferred. It always affords me great pleasure to be present at the annual meetings of our association, for apart from the pleasures enjoyed by the friendly intercourse of those engaged in a common cause, these meetings are productive of much good in disseminating useful knowledge and in elevating and advancing the interest of our profession, the meeting together and discussing of our differences evolve new ideas and better methods, and no member can fail to gain from them information that will be of daily use in his practice, and that will better equip him for the discharge of duties in ministering to suffering humanity. It is, therefore, gratifying to every one interested in dentistry to note the increasing interest manifested in this organization which has already been largely instrumental in correcting our grievances and dignifying the importance of our position among the learned professions. When I stop and allow my thoughts to revert to the first meeting of this association that I attended and compare it with the last two or three meetings, it makes me feel that the dentists have not been sleeping, but that they are well up in the march of progressive science, and have as yet no thought of lagging in the race of professional advancement. We have witnessed at this session a striking illustration of the progress in the apparently successful demonstration of the implanting of teeth by our honored guest, Dr. Herring, an operation which seems contrary to all physiological laws and would not have been attempted several years ago with any hope of success. This and like experiments and results have forced me to the conclusion that the impossible will not soon be reached in scientific investigation and invention, and that much is yet to be learned in the science to which we owe our allegiance. Another wise step in the right direction and one that will be of the utmost importance and benefit to the people as well as the profession has been taken in securing to us, by proper legislation, the privilege and right of protecting dentistry in a great measure from the imposition of ignorance and the disgrace of incompetence. I refer to the act passed by the last legislature prohibiting practice of dentistry in this state without first obtaining a license from the Board of Dental Examiners. In the matter of legislation I would suggest that an effort be made at the ensuing session of the legislature to exempt dentists from jury duty, a privilege I think we should have.

In conclusion, it rests with us to see that all quacks are banished from our ranks by enforcing the law granted us for that purpose, and thereby elevate the standard of our profession in dental education. Again thanking you for courtesies extended, I will bespeak the same kind consideration at your hands for my worthy successor.

The following gentlemen applying for membership and being approved by the Board of Examiners, were duly elected members of the Association: viz., Drs. S. S. Daniel, Spartanburg; W. M. Meador, Union; Manly Timmons, Edgefield; A. C. Strickland, Anderson; J. P. Carlysle, Greenville. Dr. Daniel being a graduate since the version of the State law regulating the practice of dentistry, was granted a license by the board to practice dentistry in the State of South Carolina.

Visiting dentists were extended the privileges of the floor during the meetings; prominently among these were Drs. John S. Thompson and R. A. Holliday, of Atlanta, Ga.

The following papers were read during the meeting, and discussed freely: "Failures," by Dr. B. H. Teague; "Micro organisms," read by Dr. J. H. Alexander, written by Dr. M. Bissell, the most aged member of the Association, now nearly or quite 85 years of age; "Amalgam" with illustrations, by Dr. Theo. Johnson, of Newberry.

"Vacuum Cavities," by A. T. Peete, of Branchville, read by Secretary Smith. Dr. G. S. Wright read a paper on "Dental Education." Dr. E. C. Ridgell read communications from Dr. Jas. Harris and C. N. Peirce; Dr. G. W. Dick, exhibited an electric dynamo, which he uses to run his electric mallet; he claims "that it is more powerful than the electric battery, is so simple on account of accuracy and certainty, is always ready for use, and has not the slightest waste excepting of course wear. The amount of the current varies according to the number of revolutions. Lights the incandescent lamp beautifully.

Dr. A. P. Johnson exhibited some specimens of crown work in the mouth of a patient, which was very creditable.

Dr. Wright mentioned that in 1884, the proceedings of the association had been printed, from the organization of the Association up to that year, in pamphlet form, including a circular on "The Teeth," the Dental Law, Constitution, By Laws, and a list of the dentists in the State. The Secretary informed the meting that several hundred of these were still on hand.

Dr. Alexander moved that ten copies of the pamphlets in the possession of the association be given each of the members applying for them, free; all over this number at the rate of 5 cents each, expenses on same to be paid by member receiving them. Carried.

Dr. Calvert applied to the association for the use of Dr. Gurley's plates for one month. Granted.

Dr. Alexander exhibited a book by Dr. Fitch, on the subject of Crown and Bridge work, illustrating this work as done many years ago, proving that all that is done now-a-days is not new.

Letters of regret were read from Drs. W. S. Brown; J. H. E. Milhous and B. H. Catching. The Association regretted their inability to be present.

The board of Dental Examiners read the following report: Resolved, "That parties tò whom permanent license are granted at this meeting and hereafter shall be entitled to membership without any other initiation fee." Carried.

The Board recommended Messrs. E. H. Webb and H. K. Smith, respectively, for the Baltimore and University ot Md., Scholarships. Endorsed.

Dr. J. S. Thompson, of Atlanta, exhibited his combination apparatus, for soldering, waxing, melting and illuminating purposes. [Note.—You can describe this as you and Dr. J. S. T. would prefer]. The thanks of the association were extended Dr. J. S. Thompson for his exhibit and illustrations, Dr. J. P. Carlysle for kind attention, and the Knights of Honor for the use of their hall,

Dr. Teague moved that hereafter the Association em ploy a stenographer at its meetings, amended by Dr. Calvert, and if necessary tax each member pro rata to pay same. Carried.

The treasurer having made sufficient collections this year, it was moved and seconded and carried that he pay all necessary expenses of the meeting, and Edward Perry for printing still due.

Dr. Teague moved that we do away with recommending beneficiaries. Carried.

Dr. J. R. Thompson was elected to fill vacancy of the Board of Dental Exrminers, occasioned by the expiration of Dr. Browns term and his request not to be re-elected. The board elected Dr. Wright as President of the Board.

The following officers were elected to serve for the ensuing year: Dr. J. H. Alexander, Camden, President; Dr. E. C. Ridgell, Bates, first vice-President; Dr. J. Ry. Smith, Williston, Second Vice-President; Dr. J. C. Oeland, Spartanburg, Corresponding Secretary; Dr. R. Atmar Smith, Charleston, Recording Secretary; Dr. G. W. Dick, Sumter, Treasurer.

The place for next meeting selected is Columbia, S. C. Time second Tuesday in May, 1889.

R. ATMAR SMITH, Rec. Secty.

—*Southern Dental Journal.*

